# Effects of a brief workplace-centered consultation for employees with musculoskeletal pain on health outcomes: a prospective cohort study

**DOI:** 10.1038/s41598-019-42387-4

**Published:** 2019-04-10

**Authors:** Harald Leiss, Miriam Hucke, Manuel Bécède, Veronika Machold-Fabrizii, Josef S. Smolen, Klaus P. Machold

**Affiliations:** 1 Department of Internal Medicine 3, Division of Rheumatology, Medical University of Vienna, Vienna, Austria; 2 Department of Internal Medicine and Gastroenterology, Hepatology, Endocrinology and Nephrology, Klinikum Klagenfurt, Klagenfurt, Austria; 3 6th Medical Department with Nephrology and Dialysis, Wilhelminenspital, Vienna, Austria

## Abstract

Musculoskeletal (MSK) diseases affect a substantial proportion of the population. Specialist consultations were offered at the workplace for people with musculoskeletal (MSK)-complaints. We analyzed data on pain and well-being as well as health economic data at baseline. Lasting effects of the consultation were analyzed at a follow-up-interview after 12 months. Baseline data of 344 individuals were available. Occupations were divided into physically highly demanding (HD) or less demanding. Women reported significantly higher pain levels and less QoL than men. Sick leave days were significantly more in HD-workers. Independent of workload, significantly higher percentages of women had cervical- and upper limb-pain than men, with significantly higher pain in upper limbs in HD-workers. 235 participants were available for telephone-follow-up. QoL and MSK-pain improved significantly. Yearly out-of-pocket spendings for treatments significantly increased. NSAID use significantly decreased, whereas use of non-drug musculoskeletal-medical-services was significantly higher after one year. Regarding MSK-symptoms in gainfully employed individuals, the study showed significantly different workload-dependent differences in QoL. Significant effects of a consultation by a MSK-specialist were shown in terms of improved MSK-pain and overall well-being. This workplace-centered consultation had significant effects on beneficial health-behavior such as decreased use of NSAID and increased engagement in gymnastics and physiotherapy.

## Introduction

Musculoskeletal (MSK) symptoms affect a substantial proportion of the population. Rheumatic and MSK diseases (MSDs) are the most frequent cause of disability-retirement and one of the most frequent causes of sick leave in Austria^1,2^. Assuming an estimated prevalence of 0.5–1% approximately 5–10 million individuals in the industrialized world are affected by rheumatoid arthritis (RA)^3^, more than 150 million suffer from osteoarthritis (OA) or any other form of degenerative MSD^4^, approximately 50 million suffer from osteoporosis and more than 350 million from spine problems^5^. The societal burden is underscored by the fact that 22% of patients suffering from RA 5 years after diagnosis were unable to work^6^ or by the significant excess mortality in osteoporotic patients^7^.

Primary care for MSDs is administered mainly by general practitioners, neurologists, orthopedic surgeons or rheumatologists. However, individuals who are actively working and not retired, frequently seek medical help or advice only when symptoms are chronically present and thus at a time when complaints have already led to loss of productivity or workability^8^. This delay is caused by neglect or negation of MSDs^9^, a lack of general information^10,11^, lack of knowledge about available therapies^12^, the availability and (geographical) proximity of specialists, or a mix thereof^8^.

Keeping people with MSDs in work is increasingly recognized as an important aim of health interventions. Several initiatives across the EU pursue this aim. Fit for Work Europe is one of these initiatives, led by The Work Foundation. The latter is a multi-stakeholder coalition driving policy and practice change regarding work and health. One aim of Fit for Work is to promote early intervention practices. Workplace health promotion in Austria is not fully defined by law, it is seen as part of the obligations in the context of occupational health protection which is regulated by federal laws and regulations defining and requiring minimal extent of health services according to size and type of company. Although research shows this approach is the most effective way of ensuring people with MSDs to enter and remain in work^13–15^, only “health-conscious” companies voluntarily offer benefits to employees in excess of the minimum statutory requirements. In order to further lower the threshold to seeking medical advice in particular for actively employed people, the “Stay active” project aimed at implementing a novel model of “low threshold” counseling regarding MSK complaints/pain in the workplace setting and during regular working hours. The intervention consisted in distributing (by mail or e-mail) to employees of the participating companies a simple MSD-recognition tool (questionnaire format). For individuals in whom the questionnaire indicated a MSK problem, specialist examination and counseling close to the workplace and thus with minimal time-loss due to a doctor’s office visit was offered. The counseling specialists encouraged early treatment or preventive measures for affected individuals. Thus, the aims of “Stay active” were to detect individuals suffering from symptoms of MSDs and to provide early recognition and initiation of treatment for (possible) MSDs with minimal threshold/delay. Because of legal requirements for data protection, the ‘Stay active’ project was dealing with participants in a strictly anonymous fashion. Therefore, in order to quantify possible effects of this early MSD-intervention/consultation, the individuals were asked to participate in the present study during the screening consultation.

In the framework of this study we collected health economic data and data on pain, well-being, and psychological factors at the time of the consultation at the workplace as well as at a follow-up interview after 12 months. The main aim of this study was to study possible effects of the short MSD-centered intervention on several outcomes related to health, sickness absence and use of health care and other services. In particular, the main outcomes were resource use for preventive or therapeutic measures as well as effects on work-absenteeism. In addition, we analyzed possible differences in degree and distribution of MSK-symptoms, general well-being and psychological factors with regard to gender and workload of the participants.

## Methods

Four Austrian companies were approached via the companies’ medical services and employee representatives. The companies were selected with the aim to reach a broad variety of occupations/professions of the employees: a large hospital, a large bank, a pharmaceutical company, and a company manufacturing heavy equipment for use in mining, transportation, and the energy industry. Due to this variety it was anticipated to reach employees with mainly sedentary work as well as workers with high physical demand. In two companies (hospital, heavy equipment manufacturer), employees’ work could be grouped into “highly physically demanding/HD” (e.g. housekeeping, welders, nurses, cooks etc.) and “less physically demanding/LD” (administrators, clerical personnel, physicians, accountants etc.) according to Austrian federal law^16^. The employees of the other two companies (a bank and a pharmaceutical company) were solely engaged in “less physically demanding” occupations.

All companies participate in an Austrian program aimed at improving health at the workplace (“betriebliche Gesundheitsförderung”), which encourages companies to offer health benefits to their employees in various ways.

The employees were asked to participate in the project via e-mail and/or paper leaflets. In this step they received a questionnaire (the Nordic questionnaire)^17^ in order to detect early signs or symptoms of MSDs. All individuals in whom the Nordic questionnaire indicated presence of a MSK problem were offered an anonymous screening consultation, i.e. the employer had no information about the identity of the participants. Because the questionnaire in this context was merely used as an alert/incentive (at least one question answered with “yes”) for the employees to accept the offer of a consultation, and because of the requirement to preserve anonymity no further analysis of e.g. prevalence of MSK symptoms or body-regions involved was performed. Appointments for the consultation were scheduled by the employees via the companies’ medical services which are required to keep any personal information confidential, i.e. hidden from the employer.

The consultation took place close to the workplace, e.g. in the offices of the companies’ medical service or physician(s) and was performed by specialists for internal medicine and rheumatology or orthopedic specialists. These specialists performed a primary check for presence of MSDs. This included a brief history, a symptom centered physical examination and, if applicable, a brief history of previous od current treatments. All individuals received a brief medical recommendation and, if possible/necessary, were given a referral to a practice/outpatient clinic specialized in MSDs (Orthopedic, Rheumatologic, Rehabilitation/Physical Medicine) in order to facilitate timely specialist treatment. The cooperating practices ensured rapid admissions upon referral under this program.

The study was supported by an unrestricted grant from by AbbVie. AbbVie furthermore covered the costs of the specialists’ consultations.

### Participants and study procedures

All individuals undergoing consultation by the MSD-specialists were asked to participate in the follow-up study. If the probands agreed to be followed up, the following data were obtained using a structured questionnaire: (i) demographic information (such as age, gender, employment status), (ii) known diagnoses/comorbidities (MSD and others), (iii) duration of complaints, (iv) intensity of MSK pain (measured on a 100 mm visual analogue scale (VAS))^18^, (v) intensity of pain related to non-MSD conditions, (vi) affected region(s), (vii) medical or non-medical treatment used so far: non-steroidal antirheumatic drugs (NSAIDs), low-(non-opioid), intermediate- (weak opioids such as tramadol), and high-potency- (strong opioids such as morphine etc.) analgesics according to WHO^19^, glucocorticoids, other medication, physiotherapy, others, (viii) number of doctor visits during the preceding year, (ix) out of pocket costs for medical treatments during the preceding year, and (x) number of days of sick leave due to MSK complaints and due to other conditions/diseases during the preceding year. If the duration of symptoms exceeded 10 years, ‘120 months’ was recorded. The specialists’ suspected diagnosis/diagnoses and recommendations for further management were recorded at the screening visit. For analysis of diagnoses, all patients’ suspected diagnoses were grouped into the following categories: RA (Rheumatoid arthritis), spondyloarthritis (SpA; this group encompasses ankylosing spondylitis, psoriatic arthritis, and arthritis associated with inflammatory bowel disease), connective tissue diseases (CTD), fibromyalgia syndrome/central sensitivity syndrome (FMS/CSS), osteoarthritis (OA), ‘other inflammatory’ MSD (such as reactive arthritis, viral arthritis, gout, etc.) and ‘other non-inflammatory’ MSD (such as herniated disc, hypermobility syndrome, spondylolisthesis, scoliosis, etc.).

In addition, participants received the following questionnaires: Euroquol-5d (EQ-5d), a general well-being questionnaire^20^, Hospital Anxiety And Depression Scale (HADS-D), a depression inventory^21^, and Functional Assessment of Chronic Illness Therapy (FACIT) Fatigue scale^22^.

For follow-up after one year, participants were contacted by telephone. The following information was collected via a structured interview by two of the authors (HL and MH) blinded to the baseline data of the participants: (i) diagnosis, (ii) therapy during the intervening months between baseline and follow-up, (iii) general wellbeing assessed by numerical rating scale (NRS), (iv) intensity of pain assessed by NRS, (v) number of doctor consultations during the intervening months since baseline visit for both MSDs and non-MSD conditions, (vi) costs for treatments (out of pocket payments not covered by health insurance), and (vii) sick leave days since inclusion in the study due to MSDs as well as sick leave days since inclusion in the study due to non-MSDs.

The study was approved by the ethics committee of the Medical University of Vienna and conducted according to the Declaration of Helsinki. Written informed consent was obtained from all participants in the follow-up study.

### Statistical analysis

All recorded data were entered into an electronic storage. Analyses were performed using SPSS Version 24 (IBM Analytics, Ehningen, Germany). Gaussian distribution was assessed by the Kolmogorov-Smirnov and Shapiro-Wilk test (α = 0.05). Homogeneity of variances was assessed using Levene’s Test. Because most parameters were not normally distributed, group comparisons for continuous data were performed by Kruskal-Wallis-Test (H-Test) or Wilcoxon-test where appropriate, for categorical variables Pearson’s χ^2^ tests were used. For post-hoc analyses Games-Howell- test was used. P-values below 0.05 were regarded as statistically significant. Where multiple comparisons were performed, Bonferroni’s correction was applied (see Tables). Because of the exploratory nature of the study, no sample size calculation was performed.

### Ethical approval, informed consent and competing interests

The corresponding author attests that all listed authors meet authorship criteria and that no others meeting the criteria have been omitted.

The study was approved by the local ethics committee and conducted according to the Declaration of Helsinki. Written informed consent was obtained from all participants in the follow-up study.

The Corresponding Author has the right to grant on behalf of all authors and does grant on behalf of all authors, a worldwide licence to the Publishers and its licensees in perpetuity, in all forms, formats and media (whether known now or created in the future), to (i) publish, reproduce, distribute, display and store the Contribution, (ii) translate the Contribution into other languages, create adaptations, reprints, include within collections and create summaries, extracts and/or, abstracts of the Contribution, (iii) create any other derivative work(s) based on the Contribution, (iv) to exploit all subsidiary rights in the Contribution, (v) the inclusion of electronic links from the Contribution to third party material where-ever it may be located; and, (vi) licence any third party to do any or all of the above.

All authors have completed the Unified Competing Interest form (available on request from the corresponding author). Klaus Machold reports personal fees from AbbVie, during the conduct of the study for serving as specialist consultant; Miriam Hucke has received an unrestricted grant by AbbVie; all others declare no support from any organisation for the submitted work, no financial relationships with any organisations that might have an interest in the submitted work in the previous three years, no other relationships or activities that could appear to have influenced the submitted work.

Klaus Machold (the lead author and the manuscript’s guarantor) affirms that the manuscript is an honest, accurate, and transparent account of the study being reported, no important aspects of the study have been omitted. No discrepancies from the study as planned did occur.

Ethical approval from the Ethics committee of the Medical University of Vienna was obtained on 31. October 2014 (EC nr. 1618/2014).

AbbVie funded the conduct of the study through an unrestricted grant and through professional fees for the consultants involved in medically counseling the participants.

The study sponsor Klaus Machold was responsible for all aspects of the protocol (conception, protocol writing, ethics committee submission, conduct, data collection, and analysis).

None of the researchers had or have any material or non-material dependency of the funding company AbbVie.

Patients were not involved in conception of the study, as it was aimed at gainfully employed people who were not necessarily suffering from any disease.

The study was not registered as clinical trial because no controlled intervention was part of this analysis.

The authors are willing to share all of the individual participant data collected during the trial, after deidentification, study protocol, statistical analysis plan, analytic code beginning 3 months and ending 5 years after article publication with researchers who provide a methodologically sound proposal to achieve aims in the approved proposal. Proposals should be directed to Klaus.machold@meduniwien.ac.at to gain access, data requestors will need to sign a data access agreement.

## Results

### Participants

The numbers of employees invited to participate in the program was 3500 in the bank, 125 in the pharmaceutical company, 2070 in the hospital, and 475 in the heavy equipment manufacturer. The proportion of “HD” employees was 26% (n = 539) in the hospital, and 37.1% (n = 176) in the heavy equipment manufacturer.

**Figure 1:**
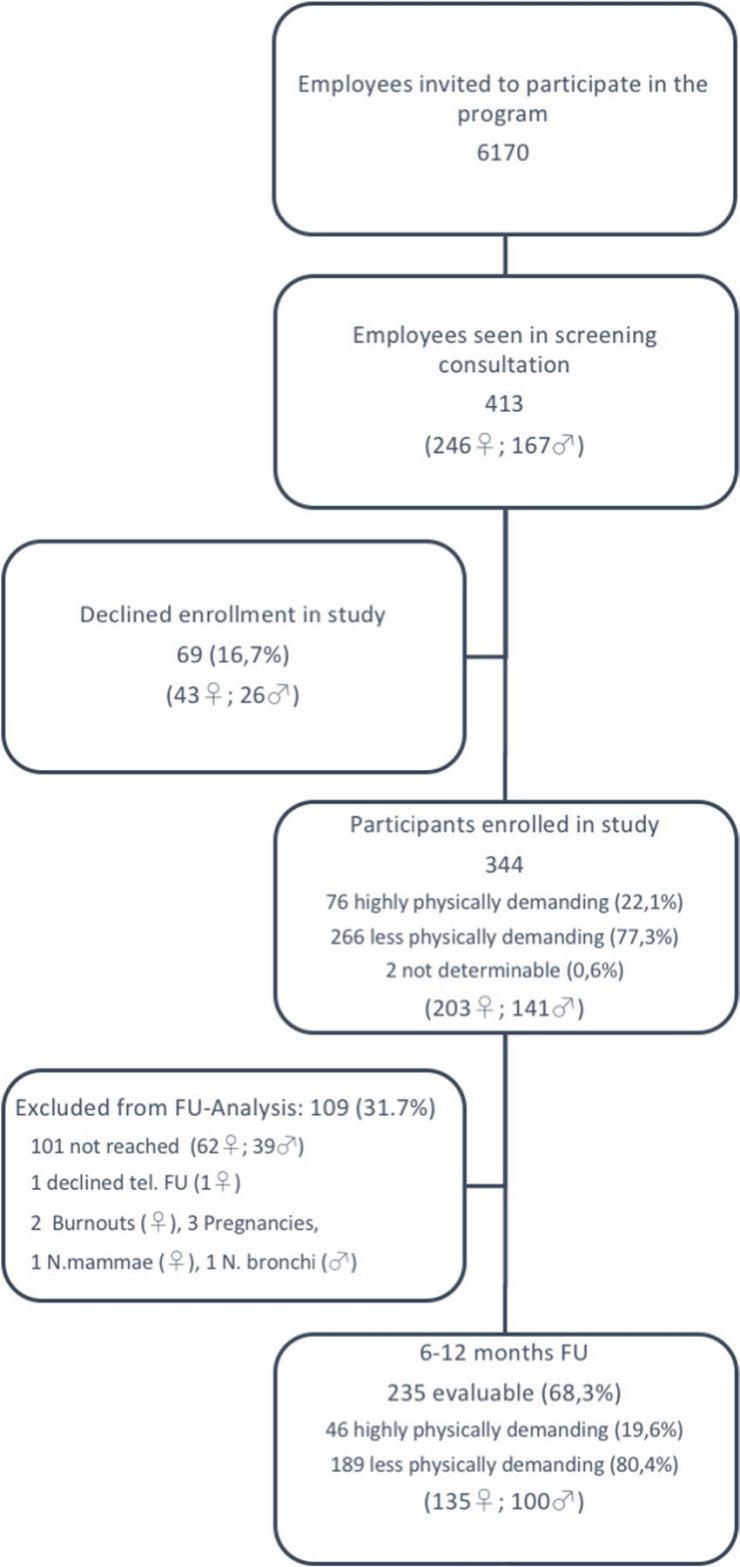
Patient disposition with regard to follow-up.

Between October 2014 and December 2016, 413 individuals (approximately 6.7% of all those who had been notified and offered the consultation), participated in the program; 344 individuals (83,3%) consented to being enrolled in the present study. 102 individuals were lost to follow-up and 7 were excluded due to interim development of medical conditions. Therefore 235 individuals were evaluable for follow-up analysis (Fig. 1). Demographic and baseline data are shown in Table 1.

**Table 1:** Baseline data for participants enrolled in the study (n = 344).

		Median	IQR	Range
Age (years)		46.85	38.02; 52.6	22.26–64.37
BMI (kg/m^2^)		24.38	22.38; 27.92	17.85–47.34
Duration of complaints (months)		24	6; 84	0–120
Pain- VAS due to MSDs (100 mm VAS)		40	20; 60	0–94
Pain-VAS due to other conditions (100 mm VAS)		0	0; 30	0–100
EQ(5d): VAS		80	60; 90	10–100
HADS-D Anxiety Score		5	3; 7	0–19
HADS-D Depression Score		3	1; 6	0–17
FACIT Score		41	33; 47	14–52
During the preceding year	Costs for treatments (€)	0	0; 26.25	0–10000
	Doctor visits due to MSDs	1	0; 2	0–60
	Doctor visits due to other complaints	1	0; 3	0–40
	Sick leave days/person due to MSDs	0	0; 0	0–140
	Sick leave days/person due to other complaints	2	0; 6	0–60

Only 71 of 344 individuals (20.9%) reported to have had to take sick leave days in the year before the consultation. Eight individuals had more than one month of sick leave because of MSDs in the year before the baseline consultation.

Before the MSD-specialist consultation no participant had been diagnosed with RA or CTD. 2 (0.6%) reported a known diagnosis of SpA, 1 (0.3%) FMS/CSS, 33 (9.6%) OA, 4 (1.2%) ‘other inflammatory’ diseases (such as reactive arthritis, viral arthritis, gout, etc.) and 109 (31.7%) ‘other non-inflammatory’ disease (such as frozen shoulder, herniated vertebral disk, cervical syndrome, spondylosis etc.). 195 (56.6%) participants reported no previous diagnosis of MSD.

The specialists’ suspected diagnoses were rheumatoid arthritis in 5 (1.5%), spondyloarthritis in 4 (1.2%), fibromyalgia/central sensitivity syndromes in 3 (0.9%), osteoarthritis in 42 (12.2%), ‘other inflammatory’ MSD-conditions in 19 (5.5%) and ‘other non-inflammatory MSD’ in 148 (43%) participants. Thus, after the specialists’ assessment, the number of individuals without suspected MSD decreased to 123 (35.7%).

The most commonly diagnosed non-MSD diseases were hypertension (n = 21 [6.1%]), thyroid diseases (n = 15 [4.4%]), diabetes (n = 12 [3.5%]), allergies (n = 7 [2%]), gastritis (n = 6 [1.7%]), solid malignancies (n = 6 [1.7%]; 5 breast cancer, 1 melanoma), headache (n = 5 [1.5%]), and asthma (n = 5 [1.5%]) (other diseases n = 8 [2.3%]). 259 (75.3%) participants did not have any other disease.

### Intergroup analysis at baseline

Of the 344 participants, 203 (59%) were female, of whom 51 (25.2%) had a highly physically demanding (HD) occupation. Of the 141 male participants 25 (17.9%) had a HD occupation.

Significantly more female participants reported pre-existing medically diagnosed ‘other non-inflammatory’ MSK conditions than men (n = 80 [39.4%] vs. n = 29 [20.6%]; p = 0.001). A significantly greater percentage of men reported no previous diagnosis of any kind of MSD (n = 95 [67.4%] vs. n = 99 [48.8%]: p = 0.001).

For further analysis of baseline data, participants were grouped according to gender and HD and LD occupations resulting in 8 “subgroups” which were compared to each other (Table 2). Women reported less quality of life (EQ-5d scale) compared with men, HD working women had the least quality of life and LD working men indicated the highest values on the EQ-5d scale. This group also reported to have the least MSK pain on the VAS scale compared to the other groups. In line with other epidemiologic studies, women reported significantly higher levels of pain than men. The number of sick leave days as well as the number of individuals who reported having taken at least one sick leave day during the preceding year due to MSD conditions were quite low overall, with a statistically significantly higher number in HD workers. Likewise, HD workers had consulted a physician for MSD-complaints during the preceding year significantly more frequently than LD workers, and women reported about twice as many physician visits because of MSDs compared with men.

**Table 2:** Intergroup analysis at baseline.

Features	Subgroups Median (IQR)								P by Kruskal-Wallis-H ANOVA	Intergroup-comparisons (p-value)							
	Female (n = 203)	Male (n = 141)	LD (n = 266)	HD (n = 76)	Female LD (n = 152)	Female HD (n = 51)	Male LD (n = 116)	Male HD (n = 25)		Female vs. male	LD vs. HD	Female LD vs. Female HD	Female LD vs. Male LD	Female LD vs. Male HD	Female HD vs. Male LD	Female HD vs. Male HD	Male LD vs. Male HD
Age (years)	48.13 (41.95; 52.61)	45.78 (36.97; 52.66)	46.54 (37.88; 52.62)	48.31 (42.53; 52.53)	47.44 (41.20; 52.61)	49.40 (44.21; 52.62)	45.25 (36.90; 52.69)	46.87 (37.41; 52.76)	0.338	●	●	●	●	●	●	●	●
BMI (kg/m^2^)	23.38 (21.30; 25.76)	25.02 (23.51; 28.83)	24.15 (22.25; 27.55)	24.98 (22.82; 29.84)	23.39 (21.30; 25.88)	23.08 (21.13; 25.33)	24.69 (23.07; 28.40)	27.91 (24.57; 30.51)	**0.001**	**0.001**	0.190	0.997	0.104	0.008	0.715	0.133	0.240
Duration of complaints (months)	24 (9; 120)	21 (2; 71.5)	24 (6; 84)	36 (6; 120)	24 (9; 84)	48 (6; 120)	24 (1; 72)	60 (12; 120)	0.095	●	●	●	●	●	●	●	●
Pain- VAS due to MSDs (100 mm VAS)*	48 (25; 61)	30 (20; 50)	35 (29; 57)	50 (23; 69)	40 (29; 60)	50 (20; 70)	30 (20; 50)	50 (30; 68)	**0.001**	**0.001**	0.011	0.853	**0.002**	0.946	**0.006**	1.000	0.057
Pain-VAS due to other conditions (100 mm VAS)*	0 (0; 4)	0 (0; 3)	0 (0; 28)	15 (0; 50)	0 (0; 30)	20 (0; 50)	0 (0; 20)	10 (0; 30)	0.115	●	●	●	●	●	●	●	●
EQ(5d): VAS**	75 (55; 90)	80 (70; 90)	80 (65; 90)	70 (55; 80)	77 (60; 90)	70 (46; 80)	80 (70; 90)	80 (70; 86)	**0.003**	**0.003**	0.021	0.298	0.021	0.706	**0.003**	0.131	0.938
HADS-D Anxiety Score^+^	5.5 (3; 8)	4 (2; 7)	5 (3; 7)	5 (2.25; 8)	5 (2.75; 8)	7 (3; 9)	5 (3; 7)	4 (2; 6)	0.055	●	●	●	●	●	●	●	●
HADS-D Depression Score^+^	3 (1; 6)	3 (1; 5.5)	3 (1; 5)	4 (1; 7)	3 (1; 5)	4 (1; 8)	3 (1; 6)	3 (1; 5)	0.093	●	●	●	●	●	●	●	●
FACIT Score^#^	39 (31; 46)	42 (36.6; 47)	41 (34.66; 47)	38 (30.25; 44)	41 (33; 47)	35 (29; 42)	42 (36; 47)	44 (36.5; 47)	**0.002**	**0.006**	0.035	0.084	0.206	0.544	**0.001**	0.030	0.994
Out of pocket costs for treatments during preceding year (€)	0 (0; 102.5)	0 (0; 0)	0 (0; 50)	0 (0; 25)	0 (0; 187.5)	0 (0; 27.5)	0 (0; 0)	0 (0; 10)	0.104	●	●	●	●	●	●	●	●
Doctor visits due to MSDs	1 (0; 2)	0 (0; 2)	0 (0; 2)	1 (0; 3)	1 (0; 2)	2 (0.75; 3)	0 (0; 2)	1 (0; 2.5)	**0.001**	**0.003**	**0.002**	0.825	0.125	0.419	0.035	0.126	0.946
Doctor visits due to other complaints	1 (0; 3)	1 (0; 3)	1 (0; 3)	1 (0; 3)	1 (0; 3)	1 (0; 3)	1 (0; 3)	0 (0; 2)	0.637	●	●	●	●	●	●	●	●
Sick leave days due to MSDs	0 (0; 0)	0 (0; 0)	0 (0; 0)	0 (0; 10)	0 (0; 14)	0 (0; 0)	0 (0; 0)	0 (0; 1)	**0.012**	0.044	**0.001**	0.204	0.200	1.000	0.010	0.260	0.429
Sick leave days due to other complaints	2 (0; 6.5)	2 (0; 5)	3 (0; 5)	0 (0; 8)	3 (0; 5)	0 (0; 9)	2.5 (0; 5)	0 (0; 7)	0.653	●	●	●	●	●	●	●	●

Data from the subgroups are shown in the left side of the table. If analysis by Kruskal-Wallis ANOVA indicated significant intergroup differences (10^th^ column from left), pairwise intergroup comparisons were done (right side of the table). For clarity, p-values are omitted in case of non-significance in the initial test. Due to significant differences of variances between groups, post-hoc group comparisons were done by Games-Howell test; *Pain-VAS on a scale from 0–100, 0 denotes no pain, 100 denotes worst imaginable pain; **EQ(5d): VAS on a scale from 0–100, 0 implicating the worst possible state of health, 100 implicating the best possible state of health; ^+^HADS-D-Anxiety and -Depression-Score, both ranging from 0–21, the higher the score, the worse are anxiety or depression; ^#^FACIT-Score ranging from 0–52, the higher the score, the better the quality of life; for multiple comparisons Bonferroni’s correction was applied and significance threshold was set at 0.00625; significant differences are indicated in bold.

On the FACIT fatigue scale women had a significantly worse score compared to men with no other significant differences in the various intergroup-comparisons. Both the HADS-D anxiety- as well as HADS-D depression score did not show significant differences in the intergroup analysis.

Regarding the HADS-D depression score and the FACIT score HD working participants also showed worse scores overall which, however, did not reach level of significance. Likewise, no significant intergroup differences were apparent regarding duration of complaints, pain rating due to other reasons than MSDs, number of medical consultations because of other reasons than MSDs, sick leave days due to other diseases than MSDs and out of pocket payments (Table 2).

With regard to painful body regions and independent of the workload, women showed significantly higher percentages of cervical- and upper limb-pain issues than men. There were significant differences between LD and HD regarding pain in upper limbs. Lower back pain was distributed equally among the groups. HD-working women additionally complained significantly more frequently about lower limb pain (Table 3).

**Table 3:** Distribution of pain and body regions at baseline.

Features	Subgroups Number of participants (%)								P by Chi^2^	Intergroup-comparisons (p-value*)							
	Female (n = 203)	Male (n = 141)	LD (n = 266)	HD (n = 76)	Female LD (n = 152)	Female HD (n = 51)	Male LD (n = 116)	Male HD (n = 25)		Female vs. male	LD vs. HD	Female LD vs. Female HD	Female LD vs. Male LD	Female LD vs. Male HD	Female HD vs. Male LD	Female HD vs. Male HD	Male LD vs. Male HD
Lower back pain	95 (47.0)	68 (49.6)	123 (46.9)	39 (52)	65 (43.3)	29 (56.9)	58 (51.8)	10 (41.7)	0.268	●	●	●	●	●	●	●	●
Upper back pain	71 (35.1)	34 (24.8)	75 (28.6)	29 (38.7)	51 (34.0)	20 (39.2)	24 (21.4)	9 (37.5)	0.057	●	●	●	●	●	●	●	●
Cervical pain	131 (64.9)	57 (41.6)	144 (55.0)	43 (57.3)	96 (64.0)	35 (68.6)	48 (42.9)	8 (33.3)	**0.001**	**0.001**	0.792	0.612	**0.001**	0.007	**0.002**	**0.006**	0.495
Shoulder pain	121 (59.9)	63 (46.0)	139 (53.1)	44 (58.7)	87 (58.0)	34 (66.7)	52 (46.4)	10 (41.7)	0.040	●	●	●	●	●	●	●	●
Upper limb pain	82 (40.6)	28 (20.4)	75 (28.6)	34 (45.3)	53 (35.3)	28 (54.9)	22 (19.6)	6 (25.0)	**0.001**	**0.001**	0.008	0.020	**0.006**	0.363	**0.001**	0.024	0.582
Lower limb pain	100 (49.5)	79 (57.7)	130 (49.6)	49 (65.3)	65 (43.3)	35 (68.6)	65 (58.0)	14 (58.3)	**0.007**	0.151	0.018	**0.002**	0.024	0.191	0.227	0.440	1.000

Data from the subgroups are shown in the left side of the table. If initial analysis by Chi2 test indicated significant intergroup differences (10^th^ column from the left), pairwise intergroup comparisons were done (right side of the table). For clarity, p-values are omitted in case of non-significance in the initial test. *Fisher’s exact test; for multiple comparisons Bonferroni’s correction was applied and significant threshold was set at 0.00625; significant differences are indicated in bold.

### Follow-up

**Figure 2:**
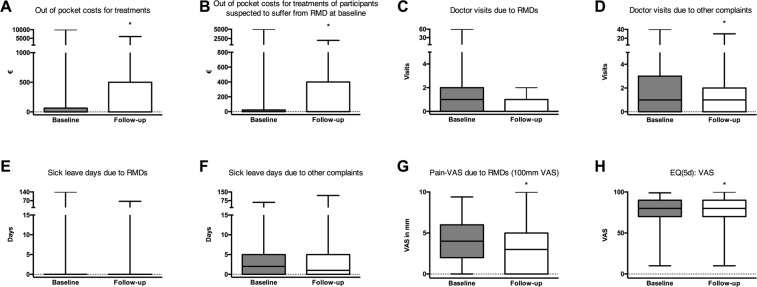
Comparison of baseline to 12 months. 235 participants with baseline and follow up data were included in the analysis. Bars show mean with range. (**A**) Changes is out of pocket costs for treatments during the last 12 months not covered national health insurance. (**B**) Changes is out of pocket costs for treatments during the last 12 months of participants suspected to suffer from MSD at baseline not covered by national health insurance. (**C**) Doctor visits due to MSDs during the last 12 months. (**D**) Doctor visits due to other complaints during the last 12 months. (**E**) Sick leave days due to MSDs during the last 12 months. F, Sick leave days due to other complaints during the last 12 months. (**G**) Pain-VAS on a scale from 0–100 during the last 12 months. 0 denotes no pain, 100 denotes worst imaginable pain. (**H**) EQ(5d): VAS on a scale from 0–100 during the last 12 months. 0 indicating the worst possible state of health, 100 indicating the best possible state of health; Wilcoxon signed-rank test was used for statistics.

For follow-up by telephone interview 235 participants (68%) were available. When asked to verbally rate their general wellbeing on a 0–100 scale (analogous to the EQ-5d scale), participants reported a significant improvement. Likewise, the rating of pain due to MSDs improved. In parallel, yearly out of pocket payments significantly increased at follow-up (Fig. 2). This was observed in all participants, regardless whether or not they were suspected by the specialist during the workplace assessment to suffer from MSDs. Days of sick leave and the number of medical consultations for MSD or non-MSD conditions per participant did not differ significantly during follow-up from the period before the consultation. During the year before the consultation at baseline, 44 of the 235 individuals for whom follow-up data were available had at least one sick-leave day due to MSD-complaints (18.7%), during the ensuing year, this number dropped to 34 (14.5%; p = 0.2150).

The proportion of participants using NSAIDs decreased significantly from 28.1% to 17.4%, whereas we found significantly higher rates of use of conservative MSK medical services (physiotherapy, gymnastics/sports, physical therapy and complementary/alternative methods), 22.1% vs. 70.2% using any type of these methods (Table 4).

**Table 4:** Comparison of medical or non-medical treatments used by the participants between baseline and 12 months’ follow-up.

Treatments	Baseline	Follow-up	p-value*
NSAID	66 (28.1)**	41 (17.4)	**0.002**
WHO II***	1 (0.9)	2 (0.9)	—
WHO III****	0 (0)	0 (0)	—
Physiotherapy	18 (7.7)	58 (24.7)	**0.001**
Gymnastics/Sports	6 (2.6)	55 (23.4)	**0.001**
Physical Therapy	29 (12.3)	102 (43.3)	**0.001**
Alternative/complementary methods	16 (6.8)	31 (13.2)	**0.018**
Any type of MSK medical service	52 (22.4)	165 (70.2)	**0.001**
Surgery in the previous/intervening year	—	6 (2.6)	—

235 participants with baseline and follow up data were included in the analysis. Data are numbers of participants (%); *McNemar-Test; **number of users (%); ***weak opioids such as tramadol; ****strong opioids such as morphine etc.

Intergroup analysis confirmed the results as described above and did not show further significant differences between gender or physical workload groups.

Because for follow-up only 235 participants’ data (68.3%) were available, we performed a sensitivity analysis in which all missing data were set as “unchanged”, with no effect on the outcomes (i.e. all the differences remained significant).

## Discussion

In this study, we assessed the effects of short MSD-intervention/consultation on several outcomes related to health, sickness absence and use of health care and other services. The employees were offered an intervention which consisted of a brief consultation by a specialist for MSDs (Rheumatologist or Orthopedic surgeon) very close to their workplace, i.e. with minimal waiting time both for the appointment as well as for the consultation itself. The consultation was offered to those out of more than 6000 employees of the participating companies, who described MSK-problems in the Nordic questionnaire. Employees were engaged in a variety of professions with a broad variety of physical demand on the individual, from very low (purely clerical work) to very high (cooks, cleaning personnel, welders, mechanics etc.). Therefore, we have captured a variety of physical symptoms in people with a broad range of physical workload with regard to location/body region as well as intensity of pain and quality of life.

The main outcomes of the study, in particular costs and types of treatment/therapy used by the participants appear to have been indeed, at least in part, been influenced by this comparatively simple and brief intervention. Out-of-pocket costs for therapeutic measures were significantly higher for the participants during the year following the consultation as compared to the year before the consultation. Furthermore, significantly more people engaged in active physiotherapy as well as in gymnastics/sports during the year following the consultation compared to the preceding year. Although the percentages still indicate that only a minority of the participants changed to a more active health behavior, the increases were remarkable: more than three times as many people took up physiotherapy, and more than nine times as many indicated to engage in sports/gymnastics. In contrast to this, a significantly lower percentage of the employees used NSAIDs (17.4%, a drop of almost 40% compared to baseline). Part of these individuals may have exchanged use of these side-effect-prone treatments for the mentioned “healthier” measures as well as for physical therapy (thermo-, electro- and mechanotherapy) which have significantly less systemic adverse effects. Use of analgesic medication with higher potency (WHO II and III opioids) was very low overall (approximately 1% at baseline), only one individual started tramadol during the year after the consultation.

We found significant differences related to gender in the distribution of painful regions: 50% more women than men indicated to suffer from pain in the cervical region and the upper limb. Cervical pain and upper limb pain generally was more frequent in women both working under high and low physical demand than in men. In contrast, low back pain affected all groups equally. To our knowledge, this difference is here reported for the first time and we believe this observation merits exploration in more detail, in particular from an occupational medical point of view. This could particularly inform preventive measures aimed at female employees.

Numerically, pain was more frequent in the HD working, especially in the limbs. This difference, however, was not significant after conservative correction for multiple observations^23–25^. Regarding the HADS-D depression score and the FACIT score we could also show worse scores in overall for HD working participants, which did not reach level of significance.

In line with published data, women indicated lower quality of life compared with men^26^, women with HD workload having the lowest, men with LD workload indicating the highest quality of life on the EQ-5d-scale. Likewise fatigue as measured by the FACIT fatigue scale was significantly higher in women than men, again with the highest fatigue (lowest score) in women under HD workload^27^. Because the study was aimed at individuals (of both sexes) who experienced MSK pain, 100% of the participants reported MSK pain. In accordance with data from the literature^28–31^, the level of pain as evaluated by VAS was higher in women, whereas workload intensity appeared to have no significant influence on the level of pain experienced.

At baseline, a majority of the participants reported no previous diagnosis of MSD. After the specialists’ assessment, approximately two thirds of the individuals were given a tentative diagnosis of MSDs by the specialist consultants. A further analysis of correctness of these “diagnoses” was impossible due to the patient-centered approach of this study. However, the number of individuals known to suffer from an inflammatory rheumatic disease increased from 6 before the baseline consultation to 28. Thus, 22 hitherto “healthy” individuals were suspected to be in need of further rheumatological workup/care.

The feasibility of offering an easy “low threshold” access to specialist health interventions was demonstrated by this initiative. Access thresholds to rheumatological care have been shown to severely delay early diagnosis (and treatment) of inflammatory rheumatic diseases, with possible negative influence on treatment outcomes^32^. Despite this very low threshold, most individuals had a duration of complaints of more than 2 years, with only a small minority (1.7%) knowingly having inflammatory MSDs. The percentage of individuals suspected to suffer from inflammatory MSDs increased to 8.1% after the consultation (although we were not able to confirm the suspected diagnoses after one year). This demonstrates that, especially in individuals who are actively employed, the tendency to ignore possibly dangerous symptoms is still substantial.

Of more than 6000 emplyees, approciamtely 6,7% took the opportunity to receive specialists’ advice. This number may appear low, given the estimated prevalence of MSK symptoms. One explanation for the low turnout may lie in the obviously quite high “individual threshold(s)” to pay attention to symptoms: the median pain rating of the participants was 40 on a 100 mm VAS, a value regarded as qualifying for high intensity treatment, and only 25% of the participants had a VAS < 20. Therefore, the opportunity for a consultation appears to have been “by design” attractive only to individuals with at least moderate pain levels, generating some sort of “bias” towards people with higher degree of pain. Despite the readily available specialist counsel, a substantial number of individuals affected by “minor” symptoms seem to have let the offered opportunity pass. Furthermore, because such an analysis was outside the scope both of the “Stay active” project as well as of the follow-up study, we can only speculate on the overall prevalence of MSK symptoms of minor intensity.

Because it was not possible to include a comparison group (e.g. without intervention) in the study we cannot rule out “secular” effects, e.g. by only alerting employees to possible MSK problems by distributing the Nordic questionnaire (without the ensuing consultation). Another confounding factor for the interpretation of the follow-up results is that it is impossible to distinguish effects of the intervention itself on pain and medication use from the effects whatever further health services (e.g. physician visits) were used in the 12 months following the consultation.

Taken together, the effects of the brief intervention offered to the participants appear to be substantial in terms of inducing beneficial health behavior such as increase of engagement in gymnastics/physiotherapy and improvement of overall well-being. These effects may be brief as we have not extended the study beyond one year. Still, the significant benefits seen may serve as an in incentive to establish and if possible, repeat such initiatives in the workplace setting on a broader scale.
